# Evaluating different interrow distance between corn and soybean for optimum growth, production and nutritive value of intercropped forages

**DOI:** 10.1186/s40781-017-0158-0

**Published:** 2018-02-05

**Authors:** Jeongtae Kim, Yowook Song, Dong Woo Kim, Muhammad Fiaz, Chan Ho Kwon

**Affiliations:** 1 Department of Animal Science, Kyungpook National University, 2559, Gyeongsang-daero, Sangj-si, 37224 South Korea; 2 Department of Livestock Production and Management, Arid Agriculture University, Rawalpindi, 46300 Pakistan

**Keywords:** Interrow distance, Seeding rows, Corn-soybean and intercropping forage

## Abstract

**Background:**

Maize fodder is being used as staple feed for livestock but it lacks protein and essential amino acids; lysine and tryptophan. Intercropping maize with leguminous soybean crop is promising technique under limited land resources of South Korea but it can only give considerable advantages when adequate distance is provided between corn and soybean rows. Main aim of present study was to find-out adequate distance between corn and soybean seeding rows for optimum growth, yield and nutritive value of intercropped forage.

**Methods:**

Different interrow distances between corn and soybean were evaluated under four treatments, viz. 1) Corn sole as positive control treatment 2) Zero cm between corn and soybean (control); 2) Five cm between corn and soybean; 3) 10 cm between corn and soybean, with three replicates under randomized block design.

**Results:**

Findings depicted that height and number of corn stalks and ears were similar (*P* > 0.05) among different treatments. Numerically average corn ear height was decreased at zero cm distance. Dry matter percentage in all components; corn stalk, corn ear and soybean was also found not different (*P* > 0.05) but dry matter yield in component of corn ear was lower (*P* < 0.05) at zero cm distance as compared to that of 5 and 10 cm interrow distances. In case of nutritive value, total digestible nutrient yield in intercropped corn was also found lower (*P* < 0.05) at zero cm distance than that of 5 and 10 cm interrow distances between corn and soybean seeding rows. Substantial decrease in dry matter yield of maize ear at zero cm distance might be attributed to factor of closed interrow spacing which made interplant competition more intensified for light interception, necessary for photosynthetic activity. Lower dry matter yield in ear also reduced total digestible nutrients in intercropped maize because it was determining factor in calculation of digestible nutrients. The optimum yield and nutritive value of forage at wider interrow distance i.e. 5 cm between corn and soybean might be due to adequate interseed distance.

**Conclusion:**

Conclusively, pattern of corn and soybean seeding in rows at 5 cm distance was found suitable which provided adequate interrow distance to maintain enough mutual cooperation and decreased competition between both species for optimum production performance and nutritive value of intercropped forage.

## Background

In connection to achievement of high economic growth in Republic of Korea, consumption pattern is massively shifted towards meat and dairy products. Accordingly, role of livestock industry has become more prominent as an attempt to meet increasing domestic demand. Therefore, livestock production reaches 40.2% of total Agro-forestry production [1, 2]. Consequently, livestock population of beef cattle, pigs, chicken, ducks and dairy cattle is reached to 2742, 10,355, 101,014, 10,705 and 402 thousand heads, respectively during second quarter of year 2016 [3]. This considerable number of livestock requires ample amount of feeding resources in country through import or local production. Unfortunately, self- sufficiency in production of feeding resources for livestock is quite low due to limited cultivatable land and use of traditional cropping techniques.

Maize is worldwide renowned as king of cereal fodders which can be used as major feed ingredient in livestock. It is good source of carbohydrates and provides 60% energy and 90% starch in animal’s diet [4]. Farmers prefer to cultivate maize as staple feed for their animals because it can be easily processed and preserved for silage. However, maize forage lacks protein and essential amino acids; lysine and tryptophan [5]. Legumes are known to be an excellent source of protein and might be used to cover protein deficiency of cereals [6]. In attempt to improve the nutritive value of maize, intercropping maize with soybean is getting recognized in Republic of Korea by farmers as a promising technique under limited land resources [7]. Mixture of nitrogen fixing leguminous and none fixing cereal crop would gave more productivity than mono-cropping [8] through biculture rhizobial symbiosis [9]. Intercropped legumes can fix nitrogen from atmosphere and do not compete with companion maize crop for nitrogen resources [10] making cereal legume biculture system more superior than mono-cropping [11].

Main aim of intercropping strategy has been to utilize resources such as distance, light and nutrients efficiently [12] for enhancing forage quality as well as quantity [13]. When two independent crops are grown together, plants of each component need enough distance in facilitating cooperation and competition between crops. Adequate interseed distance is quite important to be considered in preliminary spatial arrangements because spatial arrangement are determining factor for optimum productivity in corn-soybean intercropping [14]. Any change in hierarchy and spatial pattern can greatly influence productivity of intercropping strategy [15]. The literature regarding optimum interseed distance between corn (Zee mays L.) and soybean (Glycine max L.) rows in intercropping system under Korean environment conditions is scanty. Therefore present study was designed with main objective to find-out adequate distance between corn and soybean seeding rows for optimum growth, yield and nutritive value of intercropped forage.

## Methods

### Location of research site

Research trial was conducted at private farm in Angang-eup of Gyeongju city in Gyeongbuk province of South Korea. Its geographical coordinates are 36°00′51.5”N 129°12′13.5″E. The comparative average temperature and total rainfall recorded during study period and last five years is given in Table 1.

**Table 1:** Comparative average temperature and total rainfall in Gyeongju city, Gyeongbuk

Climate	Year	June	July	Aug	Sep
Temp (°C)	2015	21.2	24.0	25.4	19.8
	2010–2014	21.4	25.4	25.3	20.2
Rainfall (mm)	2015	22.4	171.6	104.2	37.0
	2010–2014	90.4	170.0	243.8	82.5

Source: Korea Meteorological Administration, 2016

### Experimental treatments and design

Different interseed distances between corn and soybean seeding rows were evaluated under three research treatments, viz. 1) corn sole seeding (positive control), 2) zero cm distance between corn and soybean seeding rows (control), 3) five cm distance between corn and soybean seeding rows and 4) ten cm distance between corn and soybean seeding rows. Each treatment was replicated three times following randomized block design.

An area of land having length and width measurement (15 × 12 m) was selected and preliminary divided it equally into 3 main blocks (replicates); A, B and C. Then each block was further divided into 4 plots. Each plot had length and width (5 × 3 m). Finally, 12 plots were made available for random application of four treatments with three replicates.

### Seed and seeding

In connection with corn soybean intercropping, maize (variety named Pioneer P1184) and soybean (crossbred variety named PI483463 × Hutcheson) were sown on 15th June of 2015, whereas harvesting was furnished on 8th October of same year. The land was prepared with application of fertilizer NPK (21:17:17) at the rate of 200 kg per hectare.

Preliminary, seeding of corn was accomplished on equally distant 4 lines in each plot. In case of positive control treatment for corn, soybean seeding was not performed leaving plots for corn mono-cropping. However, soybean seeding under 2nd treatment (control) was conducted over the same rows where corn seeding was done previously to ensure zero cm distance between corn and soybean seeds. Whereas, seeding of soybean under 3rd treatment was carried out on separate parallel rows which were 5 cm distant away from corn seeding lines. The same method was followed in 4th treatment but interseed distance was fixed 10 cm between corn and soybean rows.

Mixture of Alachlor and Pendimethalin herbicides was used after completion of seeding. The management and conditions like temperature, moisture, air and lighting were kept similar and identical to all experimental treatments.

### Data collection

Height and number of maize stalk, maize ear and soybean was measured and counted on harvesting time. Maize height was taken from ground to top of plant and height of maize ear was measured from ground to the bud of ear evolved, whereas soybean height was measured from ground to top of plant. Five plants were taken randomly from each replicate for measuring data regarding height. Similarly, 2 samples from each replicate were randomly taken for dry matter and total digestible nutrient yield, initially weighed, dried in oven at 70 °C for 72 h and then again weighed after drying. The percentage of DM was just calculated using fresh yield and dry matter yield information. Total Digestible Nutrients (TDN) was calculated through following equation [16],

*Total digestible nutrient = (DM yield of maize stalk × 0.582) + (DM yield of maize ear × 0.85).*

*Where, numbers 0.582 and 0.85 are constant factors used to calculate TDN.*

### Parameters studied

Effect of varying interrow spaces between corn and soybean was investigated in terms of following parameters, viz. corn stalk height and number, corn ear height and number, soybean height and number, coupling number, dry matter percentage, dry matter yield (corn stalk, ear and soybean) and total digestible nutrients yield (corn stalk plus ear).

### Statistical analysis

Data were statistically analyzed using ANOVA technique under randomized block design through SAS 9.1.3 software. The difference among treatment means was tested through Duncan Multiple Range Test [17] and confidence level for this experiment was 95%.

## Results

### Effect of different interrow space between corn and soybean on growth performance of forage

Response of interrow distance on growth performance of mixed forage is mentioned in Table 2. The height and number of corn stalks and ears were not significantly different (*P* > 0.05) among treatments of different interseed intervals. Similarly, soybean height, soybean number and corn soybean coupling were also not different among treatments. It was depicted that numerically average corn ear height was decreased at zero cm distance between corn and soybean seeding rows, although it was not significantly different from other treatments. In case of corn sole (Positive control treatment), the corn stalk height was not different (*P* < 0.05) with intercropping corn component at any interrow space but it was tended to high at 5 cm interrow distance between corn and soybean.

**Table 2:** Effect of different interrow space between corn & soybean on growth performance of forage

Parameters (Mean ± SE)	0 cm Corn sole (Positive control)	0 cm distance Corn soybean (Control)	5 cm distance Corn soybean	10 cm distance Corn soybean
Corn stalk height (cm)	250.4 ± 4.8	244.2 ± 6.4	260.8 ± 4.8	248.3 ± 13.5
Corn ear height (cm)	75.5 ± 4.2	66.8 ± 3.6	74.2 ± 1.2	74.2 ± 5.3
Soybean height (cm)	–	46.2 ± 6.1	52.5 ± 7.7	45.4 ± 2.1
Corn stalk number (No.)	27.0 ± 2.5	27.6 ± 1.4	27.5 ± 1.5	28.0 ± 0.5
Corn ear number (No.)	26.6 ± 1.3	26.0 ± 0.5	26.0 ± 2.0	26.0 ± 1.5
Soybean number (No.)	–	49.3 ± 4.2	33.5 ± 2.5	46.0 ± 7.0
Coupling (No.)	–	18.0 ± 1.5	16.5 ± 2.5	15.3 ± 0.6

Variables having no superscripts in the same row are not different (*P* > 0.05), *SE* Standard error

### Effect of different interrow space between corn and soybean on production of mixed forage

**Fig. 1:**
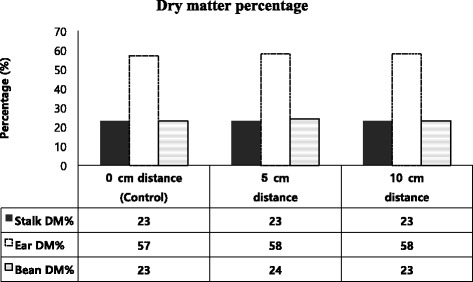
Effect of different interrow distance between corn and soybean on dry matter percentage in corn stalk, corn ears and soybean components of mixed forage (Mean ± SE). *Same colored bar variable with value over top having no superscripts are not different (*P* > 0.05)

Dry matter percentage (DM %) in all components; corn stalk, corn ear and soybean was found not significantly different (*P* > 0.05) among inter-seed distances between corn and soybean seeding rows as shown by Fig. 1. Dry matter yield (tons/ha) in corn stalk and soybean components of intercropping forage was also not significantly different among treatments of different interrow distances. However, dry matter yield in component of corn ear was found lower (*P* < 0.05) at zero cm distance as compared to that of 5 and 10 cm interrow spaces as shown in Table 3. The DMY of corn component in corn sole was not different (*P* < 0.05) with intercropping corn component at any interrow space but it was tended to high at 5 cm interrow distance between corn and soybean. However, corn ear DMY was higher (*P* < 0.05) than that of zero cm treatment but not different (*P* > 0.05) with other treatments.

**Table 3:** Effect of different interrow space between corn & soybean on dry matter yield of forage

Parameters (Mean ± SE)	0 cm distance Corn sole (Positive control)	0 cm distance Corn & soybean (Control)	5 cm interrow distance Corn & soybean	10 cm interrow distance Corn & soybean
Corn stalk DMY (ton/ha)	5.9 ± 0.4^a^	5.1 ± 0.4^a^	6.3 ± 0.6^a^	5.5 ± 0.5^a^
Corn ear DMY (ton/ha)	6.6 ± 0.5^a^	4.8 ± 0.6^b^	7.2 ± 0.03^a^	6.7 ± 0.4^a^
Soybean DMY (ton/ha)	–	0.8 ± 0.1^a^	0.7 ± 0.3^a^	0.9 ± 0.2^a^
Total DMY (ton/ha)	12.5 ± 0.9^ab^	10.7 ± 0.7^b^	14.2 ± 0.3^a^	13.1 ± 1.03^ab^

^a, b^Variables having varying superscript in the same row are different (*P* < 0.05), *SE* Standard error, *DMY* Dry matter yield

### Effect of different interrow space between corn and soybean on nutritive value of mixed forage

**Fig. 2:**
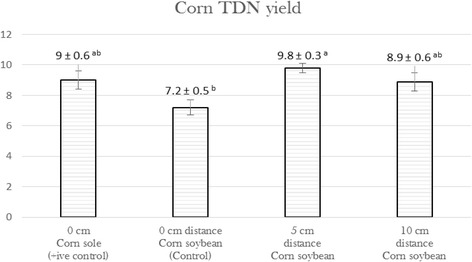
Effect of different interrow distance between corn and soybean on total digestible nutrient in intercropping corn forage (Mean ± SE). *Bar variable with value over top having different superscripts are different (*P* < 0.05)

Pattern of effect on nutritive value in terms of total digestible nutrients (TDN) yield was similar to that of dry matter yield as shown in Fig. 2. The TDN yield in component of corn (Stalk plus ear) was also found lower (*P* < 0.05) at zero cm distance than that of 5 and 10 cm interrow distances between corn and soybean.

## Discussion

The substantial reduction in dry matter yield of intercropped maize ear at zero cm interrow distance between corn and soybean in present study might be attributed to factor of close interrow spacing which consequently affect interplant competition for light interception [18–20]. The light interception could be more available at wider interrow spacing than that of close interrow distance [20–22]. Intensified interplant competition for factor of light interception might influence dry matter yield of intercropped maize ears [23] because maize yield was linearly correlated with photosynthetic output [19]. If row spacing were increased in intercropping, photosynthetic activity of forage could be enhanced [24]. In connection to comparison of corn sole with other treatments, higher tendency of intercropping corn stalk height, corn stalk DMY and significantly higher intercropping corn ear DMY under 5 cm distance treatment might be due to suitable interrow space between corn and soybean seeding lines which could fetch optimum advantage of intercropping corn with soybean through efficient rhizobial symbiosis between two intercropped species [25].

Following pattern of dry matter yield in maize ear, TDN yield in intercropped corn forage was also comparatively lower (*P* < 0.05) at zero distance between corn and soybean seeding rows than that of wider interrow spacing. The possible reason might be lesser dry matter (DM) yield in maize ear as depicted by findings of this study. The DM yield of both components of intercropped maize forage; stalk and ear were determining factor of calculating its total digestible nutrient yield as mentioned in TDN formula. Better nutritive value of intercropped corn forage in terms of TDN at wider spaced (5 cm) seeding rows between corn and soybean might be due to adequate interseed space. Previously, Yang et al. (2016) also elaborated the influence of interrow space on yield of maize forage even in maize soybean relay strip intercropping system. In that study narrow-row spacing of maize ranged from 80 to 20 cm under relay strip intercropping, the yield of intercropped maize decreased by 25.53–3.13% [26].

Maize growth could be highly sensitive to spatial arrangement [27] and interplant competition might affect growth of neighboring plants negatively [28]. Although comparative data between 5 cm and 10 cm distance treatments showed non-significant difference but distinct higher tendency in corn stalk height, corn stalk DMY and corn ear DMY and even total mixed forage DMY under 5 cm distance treatment might be due to factor that 5 cm distance could be optimum space between corn and soybean seeding lines than wider interrow space of 10 cm in this study.

In contrast to previous published literature, growth performance of plants in this study should also be decreased significantly at zero cm inter-row distance due to intensified interplant competition. However, growth parameters were not significantly (*P* > 0.05) different among various inter-seed spaces between corn and soybean due to larger variation in data. Anyhow, average corn ear height was tended to decrease at zero cm interrow distance, although it was not significantly different from other treatments. This might be considerable limitation of this study and needs further research to be conducted for authentication of adequate interrow space between in corn soybean intercropping.

## Conclusion

Conclusively, pattern of corn and soybean seeding in rows at 5 cm interrow distance was found suitable which provided adequate interplant space to maintain enough mutual cooperation and decreased competition between both species for optimum production performance and nutritive value of intercropped forage.

## Abbreviations

Bean, Soybean and DMY: Dry matter yield
DM: Dry matter
NPK: Nitrogen phosphorus potassium
SAS: Statistical analysis system
SE: Standard error
TDN: Total digestible nutrients
Temp: Temperature
